# Monthly Summary

**Published:** 1896-10

**Authors:** 


### Monthly Summary.

CAPPING PULPS.—The following method has been successfully used for a number of years, the results for the past four years having been carefully noted, during which time more than a hundred pulps have been capped by this method, each carefully watched, and only five failures recorded to date. Two of these were helpless from the beginning, having given trouble for three months, and were capped at a risk as an experiment. Two others had ached for a short time, but without inflammation. The fifth developed a pulp stone, necessitating removal of the pulp. We are persuaded that, in the field of dental operations, the capping of the dental pulp is as successful, properly performed, as the average dental operation. Gentlemen, capped pulps do live.

My method of capping is as follows: The patient rinses the mouth with as warm water as can be used comfortably, to which is added a few drops of an antiseptic. The cavity is then washed with warm water from the syringe and excavated as usual, wiping out with a small pledget of cotton saturated with chloroform. Using sterilized scissors and foil pliers, a made cap is cut "from a prescription blank" and dipped in chloroform, which quickly evaporates, leaving the paper of its original stiffness, and sufficiently sterilized. On this cap, with a small pointed sterilized instrument, is placed the smallest particle of chloro-percha to the chloroform, with fifty grains of aristol to the ounce of chloroform. This little plaster is turned over on the point of pulp exposure and gently pressed to position with the smallest piece of spunk, and a few drafts of hot air thrown on the cap, which evaporates the chloroform, leaving the cap sticking to its position. Then thin cement is flowed over it and the filling is inserted as desired.—Dr. Gordon White, in *Ohio Dental Journal*.

MASTICATING.—If we say we know how to masticate properly, we sin against light and knowledge. We had better say we do not know. I have often ask dentists how much they urge their patients to masticate properly? Some of them say they never speak to their patients about it, and very rarely have I heard one say that he gave any special information or urged his patients to masticate thoroughly. It is not only the mastication, but the thorough insalivation, that is required. Those persons who masticate their food most thoroughly have the best teeth. They have the least dyspepsia and the best nourished tissues in the body all through, and are better able to withstand all attacks of disease than those who do not masticate thoroughly. I know from observation that the majority do not masticate their food in anything like an adequate degree. I have noticed in this village a number of dentists, and I have observed that they take their meals in a few moments' time, the food not being thoroughly masticated nor thoroughly insalivated. I believe if the dentist can impress on his patient the importance and the necessity of thorough mastication, then he has done one of the greatest services for his patient that is within his power. It is better than treating the disease and conditions which we so frequently meet. It is hygiene of the mouth and the teeth, and it is for the benefit of the entire organization of the patient as well as of the teeth. The mother, the father, the nurse, and anybody in care of a child should notice it as early as three years of age, and teach it to masticate thoroughly and properly. The habit will stay with it through life, and prevent many of the ills and distresses that assail us.—Dr. Taft, in *International*.

---

IN swaging any metal I always oil my dies to prevent, as far as possible, the baser metals adhering to the plate, and before annealing wipe off all trace of the baser metals. After annealing and partial swageing, wash the plates in sulfuric acid and boil them so as to peel off the base metals. I prefer the use of cotton-seed oil for mixing modeling sand, to that of water, the steam from which caused the formation of air bubbles in the metal cast.—L. P. Haskell, in *Items of Interest*

A NEW METHOD OF DOING BLOODY OPERATIONS.—This is advised by Neudoerfer, of Vienna. For twelve years he has used peroxide of hydrogen ($H_2 O_2$) as a disinfectant and styptic. A wound treated with this fluid does not bleed during or after the operation, does not become inflamed, does not suppurate, neither is there any reaction from it. He operates as follows: The field of operation is shaved and cleansed with soap, washed and moistened with ether. Upon the instruments is poured boiling water for a quarter of an hour. The surgeon's hands and those of his assistants are thoroughly cleansed with soap and hot water, and washed with ether. No sublimate or other antiseptic comes in contact with patient, surgeon, instruments or bandages. For drying and cleaning wound are used gauze pieces saturated with $H^2 O^2$ and well pressed out. These are held firmly upon the wound for one-fourth to one-half second. The few still bleeding vessels are tied; the whole surface is again irrigated with $H^2 O^2$ and exposed to the air one or two minutes. Draining tubes are not absolutely needed. The edges are then closed by satare. The stump is covered with gauze and absorbent cotton and secured by a bandage with moderately firm pressure.

The truth is as follows:

1. The wound is covered with a slight foam; there is a little hemorrhage as after an Esmarch bandage. Capillaries, small arteries and veins do not bleed at all, but should be secured by suture.

2. Pain is relatively slight; shock is markedly absent. The patient eats, sleeps and feels well.

3. The process of healing is ideal. When the dressing is changed on the third day for removal of drains, it is found dry, the skin is pale as if nothing had been done. The new dressing may remain from eight to fourteen days, until entire healing has taken place.

The author also refers to the value of $H^2 O^2$ in epistaxis, which may be stopped by simple atomization or touching of the nasal cavity with $H^2 O^2$. As the latter is a poison to the brain and spinal cord, the quantity used should be carefully graded.—"Int. Klinische Rundschau.—Medicine."

